# Dataset of 16S rRNA gene sequences of 111 healthy and Newcastle disease infected caecal samples from multiple chicken breeds of Pakistan

**DOI:** 10.1016/j.dib.2024.110957

**Published:** 2024-09-19

**Authors:** Aqsa Ameer, Farrukh Saleem, Ciara Keating, Ozan Gundogdu, Umer Zeeshan Ijaz, Sundus Javed

**Affiliations:** a Department of Biosciences, COMSATS University Islamabad, Pakistan; b Water & Environment Research Group, University of Glasgow, Mazumdar-Shaw Advanced Research Centre, Glasgow, United Kingdom; c Department of Engineering, Durham University, Durham, DH1 3LE, UK; d School of Biodiversity, One Health, and Veterinary Medicine, College of Medical, Veterinary and Life Sciences, University of Glasgow, Glasgow, UK; e Department of Infection Biology, Faculty of Infectious and Tropical Diseases, London School of Hygiene and Tropical Medicine, London, United Kingdom; f Department of Molecular and Clinical Cancer Medicine, University of Liverpool, Liverpool, United Kingdom; g College of Science and Engineering, University of Galway, Ireland

**Keywords:** Poultry, Gut Microbiome, Operational Taxonomic Units, Vaccination, Infection

## Abstract

The article presents a processed dataset from amplicon sequencing of the V4 region of the 16S rRNA gene to recover bacterial and archaeal taxa from the caeca of multiple chicken breeds of Pakistan. These include chicken breeds commonly raised at commercial level, Naked Neck, Black Australorp, Rhode Island Red, White Layer, and Broiler. All the breeds were challenged with Newcastle Disease Virus (NDV), with vaccination against the disease also explored. This resulted in samples belonging to four treatment groups as: Control; Vaccinated; Vaccinated and Challenged; and Non-vaccinated and Challenged. These were raised on an antibiotic free diet in a semi-controlled farming setup. 16S rRNA gene amplicon sequencing of caecal DNA from day old and mature chicken samples (22 weeks for Naked Neck, Black Australorp, Rhode Island Red and White Layer; 8 weeks for Broiler) of the four groups was performed. The paired-end reads from all the samples were quality trimmed, error corrected, and overlapped, on which unique Operational Taxonomic Units (OTUs) were obtained at 99 % similarity. Using predictive modelling, the MetaCyc functional pathways, as well as KEGG orthologs were also recovered. The generated data may be used to explore microbial interactions in gastrointestinal tract with respect to NDV vaccination and infection, together with increased understanding of chicken health and productivity.

Specifications Table

**Table:** 

Subject	Biological Sciences: Microbiology: Microbiome.
Specific subject area	Caecal microbial communities of multiple chicken breeds
Type of data	FASTA files/Tables.
Data collection	16S rRNA gene amplicon sequencing was performed targeting the V4 region (primers: 515f and 806r) using the pair-end method and sequenced on the Illumina MiSeq platform*.*
Data source location	City/Country: Islamabad/Pakistan; Latitude and Longitude: 33.6844° N, 73.0479° E*.*
Data accessibility	Repository name: ncbi, figshare Data identification number: http://dx.doi.org/10.6084/m9.figshare.25795078 Direct URL to data: https://www.ncbi.nlm.nih.gov/bioproject/?term=PRJEB65106 http://dx.doi.org/10.6084/m9.figshare.25795078
Related research article	*NA*

## 1 Value of the Data

• Data provides information about archaeal and bacterial communities harbouring the gut of various indigenous and hybrid chicken breeds
• Data is useful for comparative study of abundant gut bacterial and archaeal communities of different chicken breeds.
• Data is helpful to expand the knowledge of microbe-microbe and host-microbe interaction with respect to NDV vaccination and infection.
• The presented data can be used to investigate the role of gut microbial communities in chicken health and productivity.
• Data can serve as a baseline to compare gut microbiome of other Avian species in future studies

## 2 Background

In Pakistan, 36.9 % of the households are labelled as “food insecure” [1]. Poultry industry has a pivotal role in reducing the differences between supply and demand of animal protein for the ever-growing population of Pakistan. The poultry industry contributes ≥1.3 % to the country's GDP with the employment share of around 1.5 million (https://pakistanpoultry.org/overview-of-pakistan-poultry-industry//). Poultry industry suffers tremendous economic losses due to illnesses caused by viruses [2]. Newcastle disease virus (NDV) is a poultry and wild bird pathogen that belongs to the family *Paramyxoviridae* and regarded as endemic in many countries including Pakistan [3].

It has been established that gut microbial flora serve as a contributor of controlling disease in the host [4]. The commensal microbiota has marked effects on many pathogens by way of their colonization, immune response and the antimicrobial secretions [5]. Though, the structure of the chicken gastrointestinal microbial populations is influenced by several factors including age, breed, farming conditions etc. [6]. An appropriate understanding of chicken gastrointestinal tract microbiota is a prerequisite for promoting growth and health of poultry [7]. Therefore, the intended purpose of this study is to explore differences in chicken caecal microbiota with respect to breed type, vaccination, and infection status of chicken.

## 3 Data Description

The bioinformatics workflow along with the commands used on a Linux-type environment is given in the Supplementary_Materials.pdf. The final structure of the processed data repository is shown in Fig. 2.

Whilst there are many intermediate files generated by the workflow, which correspond to numerous steps of the pipeline, the notable files and their description are as follows:

→The main directory “vsearch_tutorial” contains two subdirectories, of which, “output” directory which contains the final files useful in downstream statistical analyses. These are:

• feature_w_tax.biom → The OTU abundance table (*n* = 111 samples x *P* = 74,049 OTUs) along with taxonomy of OTUs at seven levels (Kingdom, Phylum, Class, Order, Family, Genus, Species).
• tree.nwk →The phylogenetic tree of the 74,049 OTUs given in newick format

→The second subdirectory “q2-picrust2_output” contains a subdirectory “output” which contains abundance tables as:

• ec_metagenome.[biom/tsv] → Abundance table (*n* = 111 samples x *P* = 2913 features) given in both BIOM and tab-delimited format, and comprise of Enzyme Commision (EC) numbers (https://en.wikipedia.org/wiki/Enzyme_Commission_number) recovered using PICRUSt2 software
• ko_metagenome.[biom/tsv] → Abundance table (*n* = 111 samples x *P* = 10,543 features) given in both BIOM and tab-delimited format, and comprise of KEGG Orthologs (KOs) (https://www.genome.jp/kegg/ko.html) recovered using PICRUSt2 software
• pathway_abundance.[biom/tsv] → Abundance table (*n* = 111 samples x *P* = 487 features) given in both BIOM and tab-delimited format, and comprise of MetaCyc pathways (https://metacyc.org/) recovered using PICRUSt2 software.

## 4 Experimental Design, Materials and Methods

### 4.1 Preparation and management of rearing site

The site for the rearing of experimental birds was selected in the animal house of National Veterinary Laboratories, Islamabad, Pakistan. Disinfection of the site was done with caustic soda after dry cleaning and lime paint, followed by fumigation. Separate rooms were allocated for control, vaccinated and NDV challenged groups. The rooms were further divided according to the bird's density (giving 2–3 square feet area per bird) using meshed partitioning to provide separate space to each breed. Lightening was managed 14–16 h daily. Temperature of 27 °C- 30 °C and relative humidity between 45 and 55 % was maintained using heaters and exhaust fans. Coarsely ground sawdust was used for the bedding of flock.

### 4.2 Procurement of chicks

Local and commercial breeds of chicken including Naked Neck, Black Australorp, Rhode Island Red, White Layer, and Broiler (Ross-308), were selected for the study. One hundred and twenty five, A-grade, non-vaccinated day old mixed gender chicks of different breeds (25 per breed) were purchased from one of the leading hatcheries in Pakistan.

### 4.3 Treatment groups

Birds of each breed were divided in 4 treatment groups i.e. Control, Vaccinated, Vaccinated/Challenged and Non-vaccinated/Challenged groups. Control, vaccinated and challenged birds were reared in separate rooms. To avoid cross contamination, two workers were strictly advised to take care of challenged and un-challenged birds separately, with no sharing of lab clothing and shoes.

## 5 Feed and Vaccination

All breeds except broiler were fed initially with the commercially available starter feed (mashed) up until 6 weeks and then grower feed (crumbled) up until 22 weeks. Broiler birds were fed with starter feed in 1st week and then shifted to grower feed till the end of experimental period i.e. 8 weeks. Feed consisted of agricultural products mainly soybean and maize. Feed was rich in crude protein, vitamins and minerals and free of any hormones, aflatoxins and antibiotics. Daily cleaning and washing of feeding utensils were in practice to ensure flock health. Birds were vaccinated against NDV each month through commercially available live attenuated vaccine.

## 6 Virus Propagation

For the challenge study, NDV culture was prepared using viral suspension [8]. 8–9 days old specific pathogen free (SPF) embryonated eggs were inoculated with 0.1 ml of viral suspension by amnio-allantoic route and incubated at 38 °C and 65 % relative humidity to allow the virus to grow. Non-specific death of embryo was measured daily through candling up to 3 days. The amnio-allantoic fluid (as viral suspension) was harvested after chilling of eggs at 4 °C for 24 hrs and stored at −80 °C until use.

## 7 Haemagglutination (HA) Assay

The success of the NDV culture was determined through the agglutination assay of red blood cells (RBCs) [9].

## 8 Embryo Infectious dose_50_ (EID_50_) Determination and Challenge

Infectivity titre of NDV suspension, used for challenge, was determined using Embryo infectious dose_50_ (EID_50_), as described by [10].

Amount of virus causing infection in 50 percent of inoculated embryonated eggs (EID_50_) as determined. Briefly, ten-fold serial dilutions of the virus suspension were prepared. Fifty, 8–9 days old, SPF embryonated eggs were inoculated with 0.1 ml of NDV suspension (five eggs per dilution). After 72 h post-inoculation eggs were chilled overnight. For the confirmation of NDV infection, Amnio Allantoic Fluid (AAF) from each egg was tested by rapid haemagglutination assay. Formula used to calculate the EID_50_ index is as under.

Index= $\frac{(\%\;infected\;at\;dilution\;immediately\;above\;50░\%)-50░\%}{\left(\%\;infected\;at\;dilution\;above\;50░\%)-(\%\;infected\;at\;dilution\;immediately\;below\;50░\%\right)}$

Birds were challenged orally with the viral suspension according to the calculated dose (Table 1).

**Table 1:** Calculation of EID_50_/mL of virus suspension for challenge study.

Dilution of Inoculum	No. of eggs infected	No. of eggs not infected	Accumulated numbers- Infected	Accumulated numbers- Non infected	Total	Percentage Infected (%)
10^–1^	5	0	34	0	34	100
10^–2^	5	0	29	0	29	100
10^–3^	4	1	24	1	25	96
10^–4^	5	0	20	1	21	95.2
10^–5^	3	2	15	3	18	83.33
10^–6^	3	2	12	5	17	70.58
*10^–7^	3	2	9	7	16	*56.25
10^–8^	3	2	6	9	15	*40
10^–9^	3	2	3	11	14	21.4
10^–10^	0	5	0	16	16	0

Index = (56.25 - 50) / (56.25 – 40) = 0.38.

EID_50_ = 10^7.38^/100 ul → 10^8.38^/ml → 0.92 ml.

## 9 DNA Extraction, 16S rRNA Sequencing and Shotgun Metagenomics

**Fig. 1:**
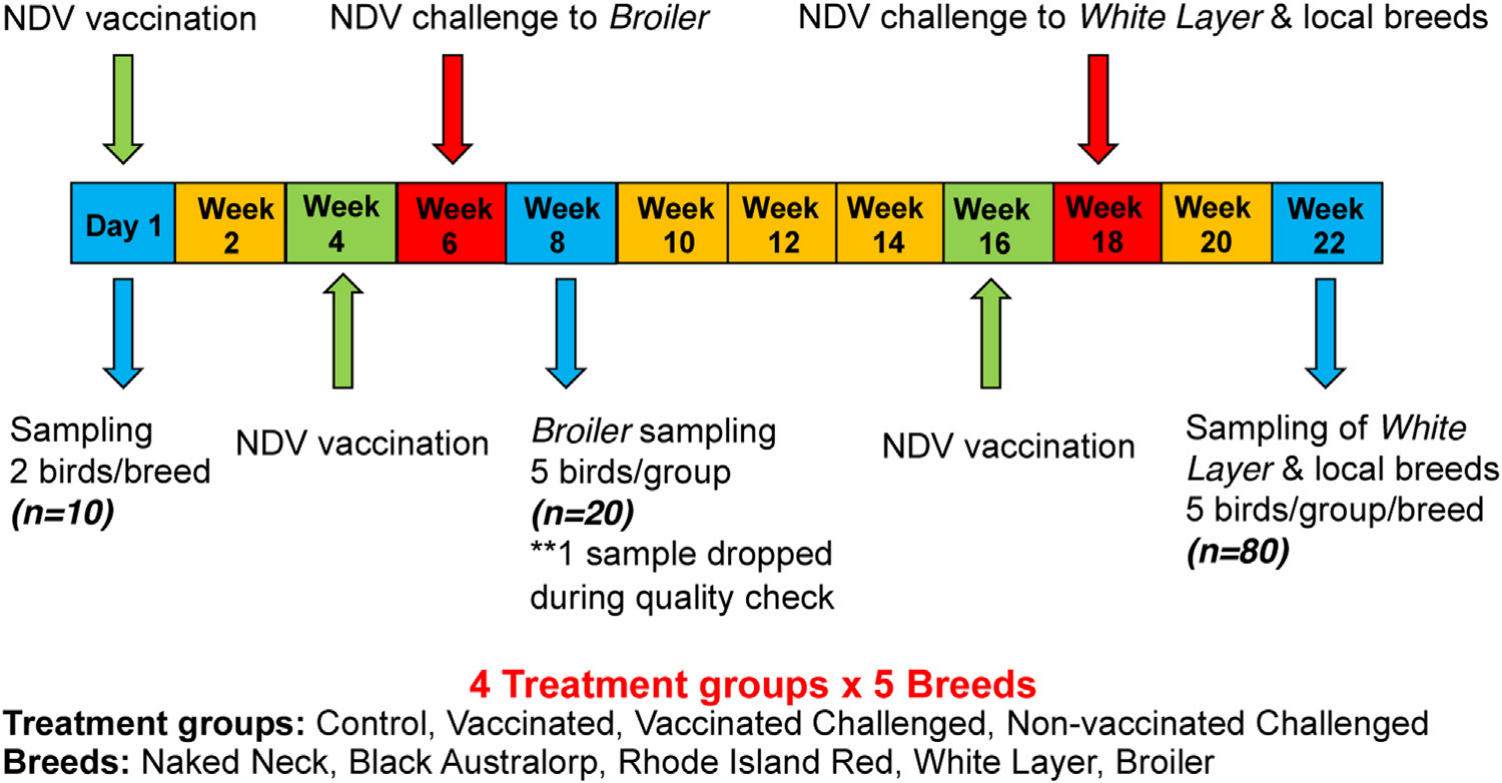
Experimental design indicating vaccination, challenges, and sampling time points along with the number of samples.

Caecal samples were collected aseptically from each treatment group (at day 1 and maturity), and maintained at −80 °C until DNA extraction. DNA was extracted using extraction kit (Invitrogen PureLink™ Microbiome DNA Purification Kit), following the manufacturer's instructions. The DNA was then shipped to Glasgow Polyomics sequencing facility for sequencing. DNA was amplified using the V4 region (515f and 806r) [11] of the 16S rRNA gene. DNA sample preparation and library quantitation has been validated using the EcoTM Real-Time PCR System. This process used PCR to selectively enrich those DNA fragments that have adapter molecules on both ends and to amplify the amount of DNA in the library. To verify the size of fragments, template size distribution was checked through running the DNA library on an Agilent Technologies 2100 Bioanalyzer. Samples were sequenced on an Illumina MiSeq machine using v2 300 standard reagent kit. The study design is given in Fig. 1.

**Fig. 2:**
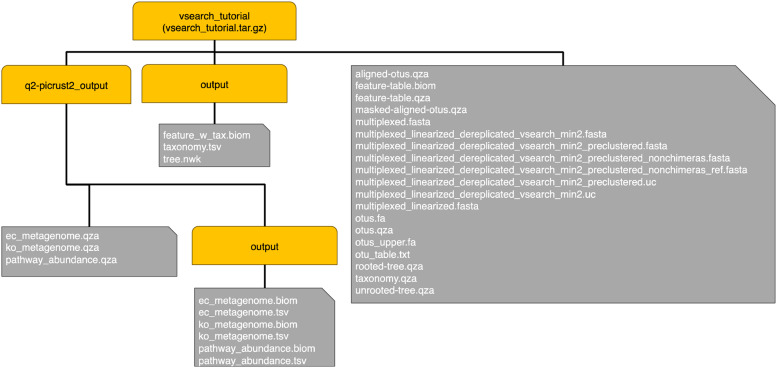
Repository structure diagram. The yellow rounded corner nodes represent directories, whilst the grey node represent files.

### 9.1 Bioinformatics

**Fig. 3:**
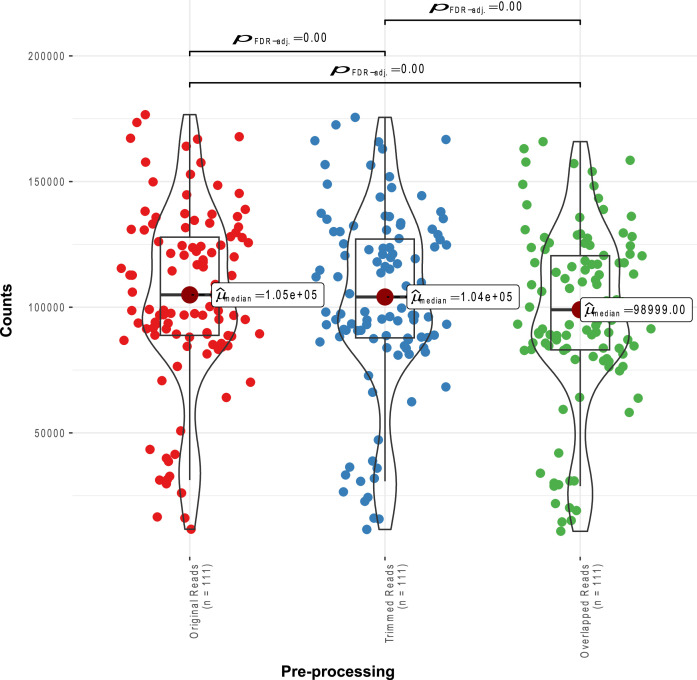
Read counts of all samples during the pre-processing steps of the bioinformatics workflow where x-axis shows different bioinformatics steps.

Abundance tables were obtained by constructing Operational Taxonomic Units (OTUs), a proxy for species level assignment using a modified workflow where the software choices for pre-processing MiSeq reads result in reducing the substitution error rates significantly [12]. Reads trimming and filtration was performed using Sickle [13] We then used BayesHammer [14] to error correct the paired-end reads. The paired-end reads were then overlapped using PandaSeq [15]. The total count of reads after these steps are shown in Fig. 3 with details given in Supplementary_Data.csv. After having obtained the consensus sequences from each sample, we used the VSEARCH pipeline [16]. All these steps are documented in https://github.com/torognes/vsearch/wiki/VSEARCH-pipeline) for OTU construction. The approach is as follows: we pool the reads from different samples together and add barcodes to keep an account of the samples these reads originate from. We then dereplicate the reads and sort them by decreasing abundance and discard singletons. In the next step, the reads are clustered, followed by removing clusters that have chimeric models built from more abundant reads (–uchime_denovo option in vsearch). A few chimeras may be missed, especially if they have parents that are absent from the reads or are present with very low abundance. Therefore, in the next step, we use a reference-based chimera filtering step (–uchime_ref option in vsearch) using a gold database (https://www.mothur.org/w/images/f/f1/Silva.gold.bacteria.zip). The original barcoded reads were matched against clean OTUs with 99 % similarity.

We have then used the recent SILVA SSU Ref NR database release v.138 [17] to assign taxonomy, and generated the rooted phylogenetic tree (using qiime phylogeny align-to-tree-mafft-fasttree) within the QIIME2 framework [18]. Furthermore, we used PICRUSt2 [19] within the QIIME environment to recover KEGG enzymes and MetaCyc pathway predictions. For this purpose, we used the parameters –p-hsp-method pic –p-max-nsti 2 in qiime picrust2 full-pipeline [https://github.com/gavinmdouglas/q2-picrust2].

QIIME2 was also used to generate a final BIOM file that combined abundance information with the new taxonomy and which along with the newly phylogenetic tree, and the meta-data was used for the downstream statistical analysis.

As a pre-processing step, we removed typical contaminants such as *Mitochondria* and *Chloroplasts*, as well as any Operational Taxonomic Units (OTUs) that were unassigned at all levels, as per recommendations given at https://docs.qiime2.org/2022.8/tutorials/filtering/. We further used R's decontam package [20] to identify and remove contaminants using blank control samples, and by employing the “Prevalence” method in it, thus giving a total of 72,835 clean OTUs for the final abundance table.

## Limitations

The insufficient number of samples per treatment group and unequal distribution of male and female birds are the limitations of the presented dataset.

## Ethics Statement

We confirm that those experiments complied with the ARRIVE guidelines and were carried out in accordance with the U.K. Animals (Scientific Procedures) Act, 1986 and associated guidelines; EU Directive 2010/63/EU for animal experiments; or the National Institutes of Health guide for the care and use of laboratory animals (NIH Publications No 8023, revised 1978). Chickens of mixed breeds were included with no association of sex on the results of study. Further the study was approved by the Ethics Review Board (ERB) at COMSATS University Islamabad (ERB No CUI/Bio/ERB-4–21/17/).

## CRediT authorship contribution statement

**Aqsa Ameer:** Conceptualization, Validation, Methodology, Formal analysis, Investigation, Data curation, Writing – original draft, Visualization. **Farrukh Saleem:** Conceptualization, Methodology, Validation, Formal analysis, Investigation, Data curation, Writing – original draft, Visualization. **Ciara Keating:** Funding acquisition, Resources, Writing – review & editing, Data curation. **Ozan Gundogdu:** Resources, Writing – review & editing, Data curation. **Umer Zeeshan Ijaz:** Software, Validation, Formal analysis, Resources, Writing – original draft, Supervision, Project administration, Funding acquisition. **Sundus Javed:** Conceptualization, Methodology, Resources, Writing – review & editing, Funding acquisition.

## Data Availability

Dataset of 16S rRNA sequences of 111 healthy and Newcastle disease infected caecal samples from multiple chicken breeds of Pakistan (Original data) (Figshare)

## Appendix Supplementary materials

Image, application 1

Image, application 2
