# Supplementary material for: Dataset of 16S rRNA gene sequences of 111 healthy and Newcastle disease infected caecal samples from multiple chicken breeds of Pakistan
Source: Data Brief. 2024 Sep 19;57:110957. doi: 10.1016/j.dib.2024.110957 (PMC11461973; doi:10.1016/j.dib.2024.110957)
Supplement: Supplementary file 1 [file mmc1.pdf]

Bioinformatics Workflow

Software and databases required (as a convention, we are using the name of software in lower case):

vsearch: Installed and available at **/home/opt/vsearch/bin/vsearch**
bayeshmmer: Installed and available at **/home/opt/SPAdes-3.13.0-Linux**
pandaseq: Installed and available under miniconda at **/home/opt/miniconda**
qiime2: Installed and available under miniconda at **/home/opt/miniconda**
sickle: Installed and available on path
bioawk: Installed and available on path
vsearch gold database: Available at **/home/opt/vsearch_GOLD_DATABASE/gold.fasta**
qiime2 trained classifier on silva 138 database: Available at **/home/opt/qiime2_databases/silva-138-99-nb-classifier.qza**
picrust2 available as part of qiime2 plugin

Organization of folders: We have each sample located in a separate folder in the directory /PATH_TO_PROJECT/sequences. Within each folder is a “Raw” folder that contains the paired-end unprocessed reads as *_R1.fastq and *_R2.fastq

```
[user@server /PATH_TO_PROJECT/sequences]$ ls -1
BA-C1
BA-C2
BA-C3
BA-C4
BA-C5
BA-D1-1
BA-D1-2
BA-NV-Ch1
BA-NV-Ch2
BA-NV-Ch3
BA-NV-Ch4
BA-NV-Ch5
BA-V1
BA-V2
BA-V3
BA-V4
BA-V5
```

BA-V-Ch1
BA-V-Ch2
BA-V-Ch3
BA-V-Ch4
BA-V-Ch5
B-C1
B-C2
B-C3
B-C4
B-C5
B-D1-1
B-D1-2
B-NV-Ch1-EXP
B-NV-Ch2-EXP
B-NV-Ch3-SD
B-NV-Ch4-SD
B-NV-Ch5-SUR
B-V1
B-V3
B-V4
B-V5
B-V-Ch1
B-V-Ch2
B-V-Ch3
B-V-Ch4
B-V-Ch5
NC1
NC2
NN-C1
NN-C2
NN-C3
NN-C4
NN-C5
NN-D1-1
NN-D1-2
NN-NV-Ch1-D

NN-NV-Ch2
NN-NV-Ch3
NN-NV-Ch4
NN-NV-Ch5
NN-V1
NN-V2
NN-V3
NN-V4
NN-V5
NN-V-Ch1
NN-V-Ch2
NN-V-Ch3
NN-V-Ch4
NN-V-Ch5
RIR-C1
RIR-C2
RIR-C3
RIR-C4
RIR-C5
RIR-D1-1
RIR-D1-2
RIR-NV-Ch1
RIR-NV-Ch2
RIR-NV-Ch3
RIR-NV-Ch4
RIR-NV-Ch5
RIR-V1
RIR-V2
RIR-V3
RIR-V4
RIR-V5
RIR-V-Ch1
RIR-V-Ch2
RIR-V-Ch3
RIR-V-Ch4
RIR-V-Ch5

```
WL-C1
WL-C2
WL-C3
WL-C4
WL-C5
WL-D1-1
WL-D1-2
WL-NV-Ch1
WL-NV-Ch2
WL-NV-Ch3
WL-NV-Ch4
WL-NV-Ch5
WL-V1
WL-V2
WL-V3
WL-V4
WL-V5
WL-V-Ch1
WL-V-Ch2
WL-V-Ch3
WL-V-Ch4
WL-V-Ch5
```

```
[user@server /PATH_TO_PROJECT/sequences]$ ls BA-C1/Raw/
BA-C1_R1.fastq  BA-C1_R2.fastq
[user@server /PATH_TO_PROJECT/sequences]$ ls BA-C2/Raw/
BA-C2_R1.fastq  BA-C2_R2.fastq
[user@server /PATH_TO_PROJECT/sequences]$ ls BA-C3/Raw/
BA-C3_R1.fastq  BA-C3_R2.fastq
```

Step 1: Trim the paired-end reads using sickle

```
[user@server /PATH_TO_PROJECT/sequences]$ for i in $(ls -d *); do cd $i;cd Raw; R1=$(ls *_R1.fastq);
R2=$(ls *_R2.fastq); cd .. ; sickle pe -f Raw/$R1 -r Raw/$R2 -o ${R1%.*}_trim.fastq -p
${R2%.*}_trim.fastq -s ${R1%.*}_singlet.fastq -q 20 -l 50 -t "sanger";cd ..; done
```

Step 2: Obtain the total number of reads after quality trimming

```
[user@server /PATH_TO_PROJECT/sequences]$ (for i in $(ls -d *); do cd $i; cat *_R1_trim.fastq; cd ..; done) | bioawk -cfastx 'END{print NR}'
11400805
```

Step 3: Run bayeshmmer on the quality trimmed reads of individual samples separately for error correction

```
[user@server /PATH_TO_PROJECT/sequences]$ for i in $(ls */ -d *); do cd $i; /home/opt/SPAdes-3.13.0-Linux/bin/spades.py -1 *_R1_trim.fastq -2 *_R2_trim.fastq -o . --only-error-correction --careful --disable-gzip-output ; cd ..; done
```

Step 4: Overlap the error corrected paired-end reads using pandaseq

In the first step, we setup the environment

```
[user@server /PATH_TO_PROJECT/sequences]$ export PATH=/home/opt/miniconda/bin:$PATH
[user@server /PATH_TO_PROJECT/sequences]$ source activate pandaseq
```

Whilst bayeshmmer has error correct the reads, with the installed version, it removes the paired end information. To make the files compatible with pandaseq, a software hack is used to put back fictitious identifiers for forward reads and reverse reads. Within pandaseq there are many choices of overlapping algorithm, the one we are interested in is simple_bayesian

```
(pandaseq) [user@server /PATH_TO_PROJECT/sequences]$ for i in $(ls */ -d); do cd $i; awk 'NR % 4==1{$0=$0" 1:N:0:GGACTCCTGTAAGGAG"}1' corrected/*R1*.cor.fastq > corrected/forward_corrected.fastq; awk 'NR % 4==1{$0=$0" 2:N:0:GGACTCCTGTAAGGAG"}1' corrected/*R2*.cor.fastq > corrected/reverse_corrected.fastq; pandaseq -f corrected/forward_corrected.fastq -r corrected/reverse_corrected.fastq -B -d bfsrk -A simple_bayesian -o 10 > $(basename ${i})".overlap.fasta"; cd ..; done
```

Now we use VSEARCH

```
(pandaseq) [user@server /PATH_TO_PROJECT/sequences]$ cd ..
```

```
(pandaseq) [user@server /PATH_TO_PROJECT]$ mkdir vsearch_tutorial
(pandaseq) [user@server /PATH_TO_PROJECT]$ cd vsearch_tutorial
(pandaseq) [user@server /PATH_TO_PROJECT/vsearch_tutorial]$
```

Step 5: Combine all the *.overlap.fasta files for individual samples together in VSEARCH/USEARCH format

```
(pandaseq) [user@server /PATH_TO_PROJECT/vsearch_tutorial]$ for i in $(ls -d ../sequences/*/); do awk -v
k=$(basename ${i}) '/^>/{$0=">barcodelabel="k";S"(++i)}1' < $i/*.overlap.fasta; done > multiplexed.fasta
```

Step 6: Check how many reads across all the samples

```
(pandaseq) [user@server /PATH_TO_PROJECT/vsearch_tutorial]$ grep -c ">" multiplexed.fasta
10749141
```

Final labels are in VSEARCH/USEARCH format: >barcodelabel=FolderName;SID. The sequences in each sample are given internal identifiers starting with S1, S2, and so on

```
(pandaseq) [user@server /PATH_TO_PROJECT/vsearch_tutorial]$ head multiplexed.fasta
>barcodelabel=BA-C1;S1
CCTACGGGCGGCTGCAGTAGGGAATCTTCCACAATGGACGCAAGTCTGATGGAGCAACGCCGCGTGAGTGAAGAAGGTCTTCGGATCGTAAAACTCTGTTGTTAG
AGAAGAACACGAGTGAGAGTAACTGTTCATTCGATGACGGTATCTAACCAGCAAGTCACGGCTAACTACGTGCCAGCAGCCGCGGTAATACGTAGGTGGCAAGCG
TTGTCCGGATTTATTGGGCGTAAAGGGAACGCAGGCGGTCTTTTAAGTCTGATGTGAAAGCCTTCGGCTTAACCGGAGTAGTGCATTGGAAACTGGAAGACTTGA
GTGCAGAAGAGGAGAGTGGAACTCCATGTGTAGCGGTGAAATGCGTAGATATATGGAAGAACACCAGTGGCGAAAGCGGCTCTCTGGTCTGTAACTGACGCTGAG
GTTCGAAAGCGTGGGTAGCAAACAGGATTAGATACCCGAGTAGTC
>barcodelabel=BA-C1;S2
CCTACGGGAGGCAGCAGTGAGGGATATTGGTCAATGGGGGAAACCCTGAACCAGCAACGCCGCGTGAGGGATGACGGCCTTCGGGTTGTAAACCTCTGTCCTCTG
TGAAGATAATGACGGTAGCAGAGGAGGAAGCTCCGGCTAACTACGTGCCAGCAGCCGCGGTAATACGTAGGGAGCAAGCGTTGTCCGGATTTACTGGGTGTAAAG
GGTGCGTAGGCGGTTTGGTAAGTCAGAAGTGAAATCCATGGGCTTAACCCATGAACTGCTTTTGAAACTATCGAACTTGAGTGAAGTAGAGGTAGGCGGAGTTCC
CGGTGTAGCGGTGAAATGCGTAGATATCGGGAGGAACACCAGTGGCGAAGGCGGCCTACTGGGCTTTAACTGACGCTGAGGCACGAAAGCATGGGTAGCGAACAG
GATTAGATACCCTGGTAGTC
>barcodelabel=BA-C1;S3
CCTACGGGAGGCTGCAGTGGGGAATATTGCACAATGGGGGAAACCCTGATGCAGCGACGCCGCGTGAGCGATGAAGTATTTCGGTATGTAAAGCTCTATCAGCAG
GGAAGAAACTGACGGTACCTGACTAAGAAGCCCCGGCTAACTACGAGCCAGCAGCCGCGGTAATACGTAGGGGGCAAGCGTTATCCGGATTGACTGGGTGTAAAG
GGAGCGCAGACGGCATGGCAAGTACGCGTGGACTGCTTTGGAAACTGTCAGGCTAGAGTGTCGGAGAGGCCAGTGGAATTCCTAGTGTAGCGGTGAAATGCGTAG
ATATTAGGAGGAACTCCAGTGGCGAAGGCGGCTTGCTGGACGATGACTGACGTTGAGGCTCGAAAGCGTGGGGAGCAAACAGGATTAGATACCCTTGTAGTC
>barcodelabel=BA-C1;S4
```

```
CCTACGGGCGGCAGCAGTGGGGAATATTGCACAATGGGGGAAACCCTGATGCAGCGACGCCGCGTGAGCGATGAAGTATTTCGGTATGTAAAGCTCTATCAGCAG
GGAAGAAACTGACGGTACCTGACTAAGAAGCACCGGCTAAATACGTGCCAGCAGCCGCGGTAATACGTATGGTGCAAGCGTTATCCGGATTTACTGGGTGTAAAG
GGAGCGCAGACGGCATGGCAAGTCTGAAGTGAAAGGCATGGGCTCAACCCGTGGACTGCTTTGGAAACTGTCAGGCTAGAGTGTCGGAGAGGCAAGTGGAATTCC
TAGTGTAGCGGTGAAATGCGTAGATATTAGGAGGAACACCAGTGGCGAAGGCGGCTTGCTGGACGATGACTGACGTTGAGGCTCGAAAGCGTGGGGAGCAAACAG
GATTAGATACCCTGGTAGTC
>barcodelabel=BA-C1;S5
CCTACGGGTGGCAGCAGTGAGGAATATTGGTCAATGGTCGGCAGACTGAACCAGCCAAGTCGCGTGAAGGAAGACGGCCCTACGGGTTGTAAACTTCTTTTGTCG
GAGAGTAAAATGCGCTACGTGTAGCGTATTGCAAGTATCCGAAGAAAAAGCATCGGCTAACTCCGTGCCAGCAGCCGCGGTAATACGGAGGATGCGAGCGTTATC
CGGATTTATTGGGTTTAAAGGGTGCGTAGGCGGCACGCCAAGTCAGCGGTGAAATACCCGGGCTTAACCCGGGAGCTGCCGTTGAAACTGACGAGCTAGAGTACA
CAAGAGGCAGGCGGAATGCGTGGTGTAGCGGTGAAATGCATAGATATCACGCAGAACCCCGATTGCGAAGGCAGCCTGCTAGGGTGAAACAGACGCTGAGGCACG
AAAGCGTGGGGATCGAACAGGATTAGATACCCGTGTAGTC
```

Step 7: Linearise FASTA file (redundant step) i.e., make the sequences appear in a single line

```
(pandaseq) [user@server /PATH_TO_PROJECT/vsearch_tutorial]$ awk 'NR==1 {print ; next} {printf /^>/ ?
"\n"$0"\n" : $1} END {print}' multiplexed.fasta > multiplexed_linearized.fasta
```

Step 8: Next we follow the steps given at https://github.com/torognes/vsearch/wiki/VSEARCH-pipeline for generating OTUs.

```
(pandaseq) [user@server /PATH_TO_PROJECT/vsearch_tutorial]$ /home/opt/vsearch/bin/vsearch --threads 40 --
derep_fulllength multiplexed_linearized.fasta --minuniquesize 2 --sizein --sizeout --fasta_width 0 --uc
multiplexed_linearized_dereplicated_vsearch_min2.uc --output
multiplexed_linearized_dereplicated_vsearch_min2.fasta
vsearch v2.3.4_linux_x86_64, 251.8GB RAM, 96 cores
https://github.com/torognes/vsearch

Reading file multiplexed_linearized.fasta 100%
4784318095 nt in 10749141 seqs, min 95, max 722, avg 445
Dereplicating 100%
Sorting 100%
5690872 unique sequences, avg cluster 1.9, median 1, max 2300
Writing output file 100%
Writing uc file, first part 100%
Writing uc file, second part 100%
1219766 uniques written, 4471106 clusters discarded (78.6%)
```

Step 9: Cluster the sequences at 99% similarity for OTUs

```
(pandaseq) [user@server /PATH_TO_PROJECT/vsearch_tutorial]$ /home/opt/vsearch/bin/vsearch --threads 40 --cluster_size multiplexed_linearized_dereplicated_vsearch_min2.fasta --id 0.99 --strand both --sizein --sizeout --fasta_width 0 --uc multiplexed_linearized_dereplicated_vsearch_min2_preclustered.uc --centroids multiplexed_linearized_dereplicated_vsearch_min2_preclustered.fasta
vsearch v2.3.4_linux_x86_64, 251.8GB RAM, 96 cores
https://github.com/torognes/vsearch

Reading file multiplexed_linearized_dereplicated_vsearch_min2.fasta 100%
552729314 nt in 1219766 seqs, min 163, max 536, avg 453
Masking 100%
Sorting by abundance 100%
Counting unique k-mers 100%
Clustering 100%
Sorting clusters 100%
Writing clusters 100%
Clusters: 194681 Size min 2, max 172055, avg 6.3
Singletons: 0, 0.0% of seqs, 0.0% of clusters
```

Step 10: Denovo chimera removal step (where you identify chimeras from the most abundant reads)

```
(pandaseq) [user@server /PATH_TO_PROJECT/vsearch_tutorial]$ /home/opt/vsearch/bin/vsearch --threads 40 --uchime_denovo multiplexed_linearized_dereplicated_vsearch_min2_preclustered.fasta --sizein --sizeout --fasta_width 0 --nonchimeras multiplexed_linearized_dereplicated_vsearch_min2_preclustered_nonchimeras.fasta
vsearch v2.3.4_linux_x86_64, 251.8GB RAM, 96 cores
https://github.com/torognes/vsearch

Reading file multiplexed_linearized_dereplicated_vsearch_min2_preclustered.fasta 100%
87996264 nt in 194681 seqs, min 164, max 536, avg 452
Masking 100%
Sorting by abundance 100%
Counting unique k-mers 100%
Detecting chimeras 100%
Found 114512 (58.8%) chimeras, 74049 (38.0%) non-chimeras,
```

```
and 6120 (3.1%) borderline sequences in 194681 unique sequences.
Taking abundance information into account, this corresponds to
494996 (7.9%) chimeras, 5388349 (85.8%) non-chimeras,
and 394690 (6.3%) borderline sequences in 6278035 total sequences.
```

Step 11: Reference based chimera removal step (a few chimeras in the previous step may be missed, especially if they have parents that are absent from the reads or are present with very low abundance, therefore we use a reference database called gold database):

```
(pandaseq) [user@server /PATH_TO_PROJECT/vsearch_tutorial]$ /home/opt/vsearch/bin/vsearch --threads 40 --
uchime_ref multiplexed_linearized_dereplicated_vsearch_min2_preclustered_nonchimeras.fasta --db
/home/opt/vsearch_GOLD_DATABASE/gold.fasta --sizein --sizeout --fasta_width 0 --nonchimeras
multiplexed_linearized_dereplicated_vsearch_min2_preclustered_nonchimeras_ref.fasta
vsearch v2.3.4_linux_x86_64, 251.8GB RAM, 96 cores
https://github.com/torognes/vsearch

Reading file /home/opt/vsearch_GOLD_DATABASE/gold.fasta 100%
7438266 nt in 5181 seqs, min 1205, max 1585, avg 1436
Masking 100%
Counting unique k-mers 100%
Creating index of unique k-mers 100%
Detecting chimeras 100%
Found 1065 (1.4%) chimeras, 72667 (98.1%) non-chimeras,
and 317 (0.4%) borderline sequences in 74049 unique sequences.
Taking abundance information into account, this corresponds to
26572 (0.5%) chimeras, 5356395 (99.4%) non-chimeras,
and 5382 (0.1%) borderline sequences in 5388349 total sequences.
```

Step 12: Generate the final otus file:

```
(pandaseq) [user@server /PATH_TO_PROJECT/vsearch_tutorial]$ /home/opt/vsearch/bin/vsearch --threads 20 --
fastx_filter multiplexed_linearized_dereplicated_vsearch_min2_preclustered_nonchimeras.fasta --threads 20
--sizein --sizeout --fasta_width 0 --relabel OTU_ --fastaout otus.fa
vsearch v2.3.4_linux_x86_64, 251.8GB RAM, 96 cores
https://github.com/torognes/vsearch

Reading input file 100%
```

```
74049 sequences kept (of which 0 truncated), 0 sequences discarded.
(pandaseq) [user@server /PATH_TO_PROJECT/vsearch_tutorial]$
```

Step 13: We need to get rid of ;size=xx from out names in the file:

```
(pandaseq) [user@server /PATH_TO_PROJECT/vsearch_tutorial]$ awk '/^>/{gsub(";.*","",$0)}1' otus.fa >
otus_backup; mv otus_backup otus.fa
```

Step 14: Generate the abundance table at 99%

```
(pandaseq) [user@server /PATH_TO_PROJECT/vsearch_tutorial]$ /home/opt/vsearch/bin/vsearch --threads 40 --
usearch_global multiplexed.fasta --threads 40 --db otus.fa --id 0.99 --sizein --sizeout --fasta_width 0 -
-qmask none -dbmask none --otutabout otu_table.txt
vsearch v2.3.4_linux_x86_64, 251.8GB RAM, 96 cores
https://github.com/torognes/vsearch

Reading file otus.fa 100%
33398477 nt in 74049 seqs, min 164, max 536, avg 451
Counting unique k-mers 100%
Creating index of unique k-mers 100%
Searching 100%
Matching query sequences: 7733488 of 10749141 (71.95%)
Writing OTU table (classic) 100%
```

The OTUs based approach has worked well, checking the summary statistics in R:

```
> abund_table<-read.csv("otu_table.txt",row.names=1,header=TRUE,sep="\t")
> abund_table<-t(abund_table)
> summary(rowSums(abund_table))
   Min. 1st Qu.  Median    Mean 3rd Qu.    Max.
   8423   58892   70719   69671   87850  121554
> dim(abund_table)
[1]   111 74049
```

Step 15: At this point, we have both otus.fa and otu_table.txt available. We now do rest of the processing in qiime2. For this purpose convert all the sequences to their uppercase representation, otherwise, qiime2 will complain

```
(pandaseq) [user@server /PATH_TO_PROJECT/vsearch_tutorial]$ bioawk -cfastx '{print
">"$name"\n"toupper($seq)}' otus.fa > otus_upper.fa
```

Enable qiime2 environment

```
(pandaseq) [user@server /PATH_TO_PROJECT/vsearch_tutorial]$ source deactivate
DeprecationWarning: 'source deactivate' is deprecated. Use 'conda deactivate'.
[user@server /PATH_TO_PROJECT/vsearch_tutorial]$ export PATH=/home/opt/miniconda/bin:$PATH
[user@server /PATH_TO_PROJECT/vsearch_tutorial]$ source activate qiime2-2019.7
(qiime2-2019.7) [user@server /PATH_TO_PROJECT/vsearch_tutorial]$
```

Step 16: Within qiime2 environment, import the OTUs in qiime2's qza format

```
(qiime2-2019.7) [user@server /PATH_TO_PROJECT/vsearch_tutorial]$ qiime tools import --type
'FeatureData[Sequence]' --input-path otus_upper.fa --output-path otus.qza
```

Step 17: Assign taxonomy using silva138 database using pre-trained naïve Bayesian classifier

```
(qiime2-2019.7) [user@server /PATH_TO_PROJECT/vsearch_tutorial]$ qiime feature-classifier classify-
sklearn --i-classifier /home/opt/qiime2_databases/silva-138-99-nb-classifier.qza --i-reads otus.qza --o-
classification taxonomy.qza
```

Step 18: Generate phylogenetic tree using mafft and fasttree

```
(qiime2-2019.7) [user@server /PATH_TO_PROJECT/vsearch_tutorial]$ unset MAFFT_BINARIES
(qiime2-2019.7) [user@server /PATH_TO_PROJECT/vsearch_tutorial]$ qiime phylogeny align-to-tree-mafft-
fasttree --i-sequences otus.qza --o-alignment aligned-otus.qza --o-masked-alignment masked-aligned-
otus.qza --p-n-threads 0 --o-tree unrooted-tree.qza --o-rooted-tree rooted-tree.qza
```

Step 19: To run picrust2, convert the otu_table.txt to BIOM format

```
(qiime2-2019.7) [user@server /PATH_TO_PROJECT/vsearch_tutorial]$ biom convert -i otu_table.txt -o
feature-table.biom --table-type="OTU table" --to-hdf5
```

Step 20: Convert BIOM format to qiime's qza format

```
(qiime2-2019.7) [user@server /PATH_TO_PROJECT/vsearch_tutorial]$ qiime tools import --input-path feature-table.biom --type 'FeatureTable[Frequency]' --input-format BIOMV210Format --output-path feature-table.qza
Imported feature-table.biom as BIOMV210Format to feature-table.qza
```

Step 21: Run picrust2 to obtain functional profiles https://github.com/picrust/picrust2/wiki

```
(qiime2-2019.7) [user@server /PATH_TO_PROJECT/vsearch_tutorial]$ qiime picrust2 full-pipeline --i-table feature-table.qza --i-seq otus.qza --output-dir q2-picrust2_output --p-threads 5 --p-hsp-method pic --p-max-nsti 2 –verbose
Warning - 596 input sequences aligned poorly to reference sequences (--min_align option specified a minimum proportion of 0.8 aligning to reference sequences). These input sequences will not be placed and will be excluded from downstream steps.
```

This produces output files in q2-picrust2_output as:

- ec_metagenome.qza - EC metagenome predictions (rows are EC numbers and columns are samples).
- ko_metagenome.qza - KO metagenome predictions (rows are KOs and columns are samples).
- pathway_abundance.qza - MetaCyc pathway abundance predictions (rows are pathways and columns are samples).

Step 22: Extract all the tables/files to be used in downstream statistical analysis

```
(qiime2-2019.7) [user@server /PATH_TO_PROJECT/vsearch_tutorial]$ cd q2-picrust2_output/
(qiime2-2019.7) [user@server /PATH_TO_PROJECT/vsearch_tutorial/q2-picrust2_output]$ qiime tools export --input-path ko_metagenome.qza --output-path output; mv output/feature-table.biom output/ko_metagenome.biom
(qiime2-2019.7) [user@server /PATH_TO_PROJECT/vsearch_tutorial/q2-picrust2_output]$ qiime tools export --input-path ec_metagenome.qza --output-path output; mv output/feature-table.biom output/ec_metagenome.biom
(qiime2-2019.7) [user@server /PATH_TO_PROJECT/vsearch_tutorial/q2-picrust2_output]$ qiime tools export --input-path pathway_abundance.qza --output-path output; mv output/feature-table.biom output/pathway_abundance.biom
(qiime2-2019.7) [user@server /PATH_TO_PROJECT/vsearch_tutorial/q2-picrust2_output]$ cd output
(qiime2-2019.7) [user@server /PATH_TO_PROJECT/vsearch_tutorial/q2-picrust2_output/output]$ biom convert -i ko_metagenome.biom -o ko_metagenome.tsv --to-tsv
(qiime2-2019.7) [user@server /PATH_TO_PROJECT/vsearch_tutorial/q2-picrust2_output/output]$ biom convert -i ec_metagenome.biom -o ec_metagenome.tsv --to-tsv
```

```
(qiime2-2019.7) [user@server /PATH_TO_PROJECT/vsearch_tutorial/q2-picrust2_output/output]$ biom convert -i pathway_abundance.biom -o pathway_abundance.tsv --to-tsv
(qiime2-2019.7) [user@server /PATH_TO_PROJECT/vsearch_tutorial/q2-picrust2_output/output]$
(qiime2-2019.7) [user@server /PATH_TO_PROJECT/vsearch_tutorial/q2-picrust2_output/output]$ cd ..
(qiime2-2019.7) [user@server /PATH_TO_PROJECT/vsearch_tutorial/q2-picrust2_output]$ cd ..
(qiime2-2019.7) [user@server /PATH_TO_PROJECT/vsearch_tutorial]$ qiime tools export --input-path rooted-tree.qza --output-path output
(qiime2-2019.7) [user@server /PATH_TO_PROJECT/vsearch_tutorial]$ qiime tools export --input-path taxonomy.qza --output-path output
(qiime2-2019.7) [user@server /PATH_TO_PROJECT/vsearch_tutorial]$ cd output
(qiime2-2019.7) [user@server /PATH_TO_PROJECT/vsearch_tutorial/output]$ sed -i s/Taxon/taxonomy/ taxonomy.tsv | sed -i s/Feature\ ID/FeatureID/ taxonomy.tsv
(qiime2-2019.7) [user@server /PATH_TO_PROJECT/vsearch_tutorial/output]$ biom add-metadata -i ../otu_table.txt -o feature_w_tax.biom --observation-metadata-fp taxonomy.tsv --observation-header FeatureID,taxonomy,Confidence --sc-separated taxonomy --float-fields Confidence
(qiime2-2019.7) [user@server /PATH_TO_PROJECT/vsearch_tutorial/output]$ ls
feature_w_tax.biom  taxonomy.tsv  tree.nwk
(qiime2-2019.7) [user@server /PATH_TO_PROJECT/vsearch_tutorial/output]$
```
